# Anthropometric indices and exclusive breastfeeding in the first six months of life: a comparison with reference standards NCHS, 1977 and WHO, 2006

**DOI:** 10.1186/s13006-015-0045-6

**Published:** 2015-06-02

**Authors:** Rosa de Fátima da Silva Vieira Marques, José Augusto de Aguiar Carrazedo Taddei, Tulio Konstantyner, Fábio Ancona Lopez, Affonso Celso Vieira Marques, Consuelo Silva de Oliveira, Josefina Aparecida Pellegrini Braga

**Affiliations:** Discipline of Pediatrics Specialties, Department of Pediatrics, Escola Paulista de Medicina/Universidade Federal de São Paulo (UNIFESP/EPM), Sao Paulo, SP Brazil; Discipline of Nutrology, Department of Pediatrics, Escola Paulista de Medicina/Universidade Federal de São Paulo (UNIFESP/EPM), Sao Paulo, SP Brazil; Department of Health Sciences, Universidade de Santo Amaro (UNISA), Sao Paulo, SP Brazil; Hospital do Servidor Público Municipal (HSPM), Sao Paulo, SP Brazil; Universidade Federal do Pará (UFPA), Belém, Brazil

**Keywords:** Growth, Breastfeeding, Infant nutrition, Growth charts, Infant

## Abstract

**Background:**

There is a gap in knowledge on the growth of children exclusively breastfed during the fifth and sixth months of life. This study aimed to assess the growth of infants who were exclusively breastfed for the first 6 months of life and compare the distributions of anthropometric measures based on the National Center for Health Statistics (NCHS, 1977) and World Health Organization (WHO, 2006) curves.

**Methods:**

Cross-sectional study that measured the weight and length of 360 healthy and exclusively breastfed infants who were enrolled in a primary care program in Belem, Brazil from October 2006 to December 2008. The children were evenly grouped into age groups from 1 to 6 months of age.

**Results:**

The mean weights were higher than the NCHS, 1977 mean weight for all of the studied groups regardless of gender and showed greater similarity to the WHO, 2006 mean weight, especially when standard deviations were considered. Regarding length, although the average length at birth was smaller, females had higher averages in the second and sixth months compared with the reference curves (*p* < 0.05).

**Conclusions:**

Exclusive breastfeeding in the first 6 months of life provides adequate physical growth, resulting in height and weight gain curves that are similar to or greater than the NCHS, 1977 and WHO, 2006 curves. The greater mean weight at the fifth and sixth months of life suggests that the second-quarter growth curves of children who are exclusively breastfed are greater than those of children who receive other types of food.

## Background

Weight and length measurements are essential for determining children’s nutritional status in the first year of life [1]. Consequently, growth charts are important tools to help health professionals determine whether a child’s physiological needs are being met whether growth and development are appropriate for age and gender [2].

Until 2006, the United States National Center for Health Statistics’ (NCHS) 1977 growth curves were the World Health Organization’s (WHO) reference for childhood and adolescent growth [3]. In 2006, the WHO released a new international standard that established rules for assessing the growth of children from birth to 5 years of age. The growth curves for children from birth to 6 months old were expanded to allow better tracking of the trajectory of weight and to evaluate the performance of lactation [1, 4–6].

A notable feature of the new WHO, 2006 growth curves was that it considered exclusive breastfeeding until the fourth month of life as the standard for determining healthy growth. Although this standard is in line with current child feeding guidelines, it contrasts with the NCHS 1977 reference, which is based solely on North American children who are fed predominantly with infant formulas [2].

Although child growth in the first year of life is affected by multiple factors, the main factor is the child's diet. Currently, the WHO, 2006 curves are the best reference standard for ideal growth, and they must be used to assess children in all countries regardless of ethnicity, diet, socioeconomic status and health status [7, 8].

Given this context and the few studies in the literature about children who receive breast milk as the sole source of nutrients for the first six months of life, especially during the fifth and sixth months [9], this study aimed to evaluate the growth of children from birth to 6 months who were exclusively breastfed (EBF) and compare their growth with the current WHO, 2006 growth curves [8] and the NCHS, 1977 curves [9].

## Methods

This was a descriptive cross-sectional study of the anthropometric measurements (weight and length) of children who were enrolled and followed for the first 6 months of life. The data were collected from October 2006 to December 2008 in a primary care setting in Belém, Pará State, Brazil.

In this particular health care location, there is a public care program for mothers and children through the age of six months to support and encourage EBF based on standard of Brazilian Ministry of Health which follows the official recommendations of WHO and the American Academy of Pediatrics [6, 10]. This program undertakes regular consultations with the binomial mother-child informing and supporting parents to practice exclusive breastfeeding. Family difficulties are managed by a multidisciplinary team according to the needs of each child. All procedures are aimed at maintaining EBF in the first six months of life.

The studied population consisted of 360 children divided into six age groups of 60 infants each 1, 2, 3, 4, 5 and 6 months (±5 days) of age, at the time of the field research. Proportional stratified sampling was used with the children's ages in months defined as the strata, assuming a confidence level of 95 % and a maximum sampling error of 4 %.

All infants enrolled at the breastfeeding promotion program and that attended the pediatric consultation during the study period were selected consecutively up to the sample target according the following inclusion criteria: were full-term, singleton birth with a birth weight of 2500 g or more, absence of perinatal morbidity, healthy and exclusively breastfed since birth up to the time of weight and length measurement.

The condition that an infant received only breast milk (including expressed breast milk or breast milk from a wet nurse), allowing receive medicine, drops and syrups (vitamins, minerals, medicines) was considered exclusive breastfeeding [10, 11].

Later, the mothers were given a pre-tested questionnaire with pre-coded questions regarding pregnancy history, delivery and postpartum conditions, neonatal history and socioeconomic conditions. Birth weight and length measurements were obtained from the child’s vaccination card or from live birth certificates. The weight (in grams) and length (in cm) were measured on the day of consultation by a trained nursing assistant. A Filizola scale certified by the National Institute of Metrology, Quality and Technology (Instituto Nacional de Metrologia, Qualidade e Tecnologia - INMETRO) with a maximum capacity of 16 kg was used to weigh the infant. The child was placed in supine position and measured with an anthropometric ruler to determine length. The adopted anthropometric techniques followed the Ministry of Health standards (Brasil [12]).

The differences between the mean weight and mean length for all of the analysed age groups were tested using Student's *t*-test for two means, and the significance level considered was *p* < 0.05. The statistical package used for data analysis was Epi Info 2000.

The field procedures were approved by the Maternal and Child Specialised Reference Unit of Public Health Secretariat of Pará (Unidade de Referência Especializada Materno Infantil da Secretaria de Saúde Pública do Pará [UREMIA-SESPA]), and the research project was approved by the Ethics Committee of the Paulista School of Medicine of the Federal University of Sao Paulo (Escola Paulista de Medicina - Universidade Federal de São Paulo [EPM/UNIFESP]). In addition, a signed informed consent form was obtained from the mothers who agreed to participate in the study.

## Results

Of the 1091 children enrolled in the exclusive breastfeeding programs, 360 mothers whose children met the inclusion criteria established by the study were selected and interviewed. The mean ages in the six strata were, respectively, 30, 61, 92, 122, 153 and 182 days, with standard deviations of five days. A total of 176 (48.8 %) infants were male, and 184 infants (51.1 %) were female.

The socioeconomic profile of the participants showed that the mothers were young, most (*n* = 212; 58.9 %) were in their teens, were primiparous (*n* = 288; 80.0 %), had up to eight years of education (*n* = 196; 54.4 %), did not work outside their homes (*n* = 327; 90.8 %) and were almost all non-smokers. Regardless of marital status, 71.4 % (*n* = 257) lived with the child's father in a stable relationship. The rate of adequate prenatal care was 99.7 % (*n* = 359) and 59.7 % (*n* = 215) had vaginal births.

The comparison of weight means and length means between the male and female children showed statistically significant differences. Regarding weight, gender differences were observed for the first (*p* = 0.0023), third (*p* = 0.0023), fourth (*p* ≤ 0.0001) and fifth (*p* = 0.0092) month groups. Regarding length, gender differences were observed at birth (*p* = 0.0291) and at 4 (*p* = 0.0015) and 5 months (*p* = 0.0077).

Table 1 presents the means and standard deviations for weight for children of both genders in this study and compares them with the reference standards. At birth and at 1 month of age, the means were similar (*p* > 0.05) for both genders. However, the means were higher than the NCHS, 1977 means in the second, third, fourth and sixth months for both genders; the means were higher than the WHO, 2006 means in the third and fourth months for males and in the second, third and sixth months for females. The means for the sample were higher than those of the standards for all groups except for the group of two-month-old males, whose means were lower than those of the WHO, 2006.

**Table 1:** Weight according to gender and age for children exclusively breastfed compared with the standards

Age (months)	Boys (mean weight, g)				Girls (mean weight, g)			
	Number	Observed (±SD)	NCHS,1977 (*p*-value)*	WHO,2006 (*p*-value)*	Number	Observed (±SD)	NCHS,1977 (*p*-value)*	WHO,2006 (*p*-value)*
Newborn	176	3272 (360)	3300 (0.532)	3300 (0.532)	184	3212 (352)	3200 (0.436)	3200 (0.436)
One	29	4446 (466)	4300 (0.152)	4500 (0.432)	31	4063 (465)	4000 (0.444)	4200 (0.134)
Two	25	5471 (406)	5200 (0.016)	5600 (0.048)	35	5353 (513)	4700 (<0.001)	5100 (0.017)
Three	27	6821 (840)	6000 (<0.001)	6400 (0.013)	33	6171 (736)	5400 (<0.001)	5800 (0.016)
Four	34	7446 (661)	6700 (<0.001)	7000 (0.008)	26	6578 (740)	6000 (0.001)	6400 (0.150)
Five	27	7680 (956)	7300 (0.114)	7500 (0.413)	33	7041 (879)	6700 (0.063)	6900 (0.494)
Six	34	8167 (694)	7800 (0.073)	7900 (0.262)	26	8086 (832)	7200 (<0.001)	7300 (0.001)

*g* Grams, *SD* Standard deviations

* Student’s *t*-test for comparison between two means

Likewise, Table 2 shows the length means and standard deviations for children of both genders, in the present study, and compares them with the reference standards. For the males, the means were similar (*p* > 0.05), except at birth. For the females, the means were higher than the NCHS, 1977 and WHO, 2006 means in the second and sixth months (*p* < 0.05).

**Table 2:** Length according to gender and age for children exclusively breastfed compared with the standards

Age (months)	Boys (mean length, cm)				Girls (mean length, cm)			
	Number	Observed (±SD)	NCHS,1977 (*p*-value)*	WHO,2006 (*p*-value)*	Number	Observed (±SD)	NCHS,1977 (*p*-value)*	WHO,2006 (*p*-value)*
Newborn	163	49.2 (1.8)	50.5 (<0.001)	49.9 (0.001)	178	48.7 (1.9)	49.9 (<0.001)	49.1 (0.006)
One	29	54.5 (1.5)	54.6 (0.828)	54.7 (0.580)	31	54.1 (1.7)	53.5 (0.118)	53.7 (0.365)
Two	25	58.4 (2.2)	58.1 (0.895)	58.4 (0.395)	35	57.9 (1.7)	56.8 (0.001)	57.1 (0.014)
Three	27	61.9 (2.2)	61.1 (0.084)	61.4 (0.264)	33	60.6 (2.5)	59.5 (0.055)	59.8 (0.168)
Four	34	64.2 (2.2)	63.7 (0.393)	63.9 (0.728)	26	62.3 (2.2)	62.0 (0.373)	62.1 (0.491)
Five	27	65.3 (2.2)	65.9 (0.202)	65.9 (0.202)	33	63.5 (2.2)	64.1 (0.068)	64.0 (0.111)
Six	34	67.5 (2.0)	67.8 (0.601)	67.6 (0.888)	26	66.8 (1.6)	65.9 (0.014)	65.7 (0.003)

*g* Grams, *SD* Standard deviations

* Student’s *t*-test for two means

**Fig. 1:**
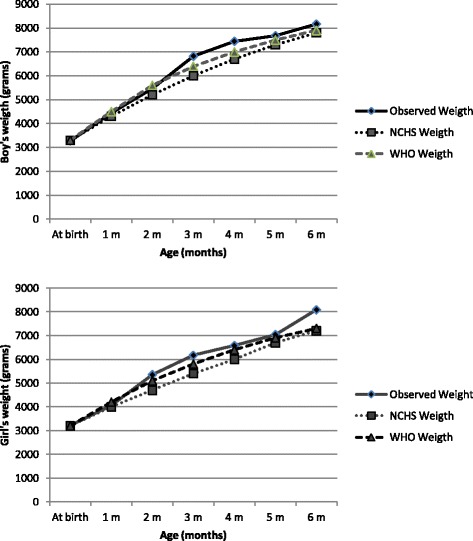
Weight means according to gender and age for children exclusively breastfed compared with the standards

**Fig. 2:**
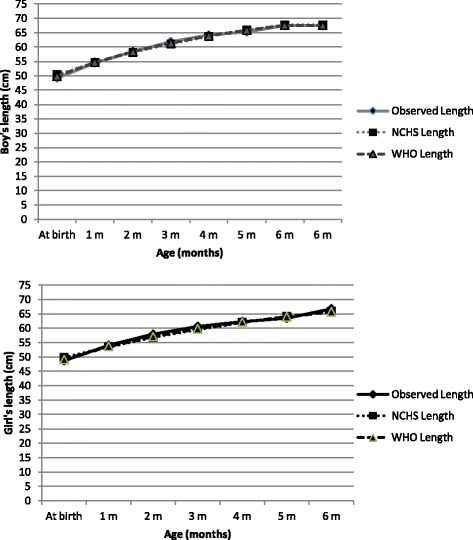
Length means according to gender and age for children exclusively breastfed compared with the standards

Figures 1 and 2 show the graphical distribution of the differences in weight means and length means between the data from this study and the reference standards to provide a visual comparison of the three curves.

## Discussion

Numerous studies in the literature evaluate the association between growth and the infant diet. However, when child age and type of food are specified, the number of studies decreases. Little research has been conducted in children who are exclusively breastfed up to 6 months of life.

Despite the adoption of strategies to encourage breastfeeding in this age group that have resulted in an increased prevalence of exclusive breastfeeding in recent decades, the prevalence rates are still far from ideal [13]. In Belém (PA, Brazil), where this study was conducted, a Brazilian representative survey found that the prevalence of exclusive breastfeeding in infants under six months of age was 56.1 %, which a higher prevalence compared with all state capitals and the Federal District [14].

Knowing the profile of those mothers who were successful with exclusive breastfeeding has been important for identifying future strategies to ensure that children have access to this natural food in the first two years of life. In our study population of exclusively breastfed infants, we observed a high number (90.8 %) of stay-at-home mothers, which corroborates the findings of another study that identified a longer duration of breastfeeding among mothers who stay at home during the first 6 months of the infant’s life [15]. However, low maternal education and maternal unemployment were also associated with an increased risk of early weaning [16].

Queiroz *et al.* [17], in a cohort study, reported that the absence of maternal cohabitation with her partner and longer EBF are variables that contributed positively and significantly to estimating the average length/age index. If we consider the EBF as a strategy to ensure proper physical growth, these results diverge somewhat from the present study’s findings because more than 70 % of the mothers who cohabited with the children's fathers continued to breastfeed their children, and their children showed adequate growth rates. Indeed, studies have shown that women who are married or living with a partner are more likely to breastfeed than women living alone [18].

Until the emergence of the new WHO, 2006 growth curves [8], the normality standards for children in the first 6 months of life had limited comparability among populations, as not all of the selected sample was EBF and newborns with low birth weights were not excluded [2, 15]. Even for the WHO, 2006 curves, the inclusion criteria required exclusive breastfeeding only until 4 months of age. Therefore, the weight and length distributions found in the present study for EBF children during the first 6 months of life may be considered to be of higher quality because they reflect growth based on the WHO and the American Academy of Pediatrics recommendations that infants in this age group be EBF.

In the United States, the Centers for Disease Control and Prevention (CDC) published a growth curve for American children in 2000 but then went on to recommend the use of the WHO, 2006 growth charts to monitor the growth of children from zero to two years of age [19]. For this reason, we did not use the CDC curves in the present study, although other studies have compared those curves with other references and populations [20].

The comparisons between curves showed that for some age groups, the weight means of the studied sample were higher than the WHO, 2006 curves, despite similar mean values at birth. The means were significantly higher among the male children in their third and fourth months and among the female children in their second, third and sixth months. Such differences most likely arise from the many advantages of exclusive breastfeeding (nutritional, immunological, psychological, emotional, economic and practicality) compared with any other form of feeding during the first 6 months of life [21].

The prevalence of exclusive breastfeeding during the first 6 months of life has increased as a result of increased support and guidance among individuals and groups. The mothers in our study were invited to participate in programs that promote breastfeeding by advocating on-demand breastfeeding and providing adequate technique training and guidance for helping the child properly latch on and suck to obtain the hind milk, which is rich in high-energy fat and thus encourages weight gain. In addition, these programs aim to inform mothers of the advantages that breastfeeding can offer mothers, their children and their families [21, 22].

Although the children in the present study had smaller lengths at birth compared with the curves that were used for comparison, they achieved similar or even higher values in later months, particularly in the second and sixth months for females. These differences can be explained by the sample size (the studied sample was smaller than the one used by WHO, 2006) and by other factors that can interfere and that were not controlled in the present study; the socioeconomic conditions of the constituent sample population, maternal nutrition, nutritional status during pregnancy and pregnancy conditions.

Children of both genders showed a mean that was very close to that expected at six months, which confirms that length during early life, although important, is not especially sensitive to changes in the nutritional status of the children in the studied age group [23]. This peculiarity may also explain the lack of large differences between the growth curves generated here and the WHO, 2006 curves.

Our findings were similar to those of Murahovschi *et al.* [24], who found curves slightly higher than those defined by the NCHS, 1997 [3]. This similarity can be explained by the fact that both studies considered only EBF children, unlike the NCHS, 1977 curves, in which artificial feeding was predominant. Furthermore, both studies focused on the assessment of children during the first months of life, unlike the NCHS, 1977 curves, which studied a broader age group and had methodological limitations related to the inclusion of children in this initial period of life.

Another study conducted in India partially agrees with the findings of the present study, stating weights were higher in infants who were breastfed compared with the NCHS, 1977 curves; however, the authors observed that length was systematically smaller [25]. In comparison, Jaldin *et al.* [26], in a Brazilian study of EBF infants up to 6 months of age, concluded that the children’s weight gain resembled the standard WHO, 2006 curves more closely than they resembled the NCHS, 1997 curves, whereas the linear growth was comparable to both curves.

These findings confirm what Augusto and Souza concluded: that exclusive breastfeeding ensures proper growth in the first two quarters of life and that the apparent changes in growth should be evaluated cautiously to avoid early weaning with the introduction of unnecessary and inappropriate complementary feeding [4].

The importance of the inclusion of EBF children in the analysis was that it made it possible to generate a standardised weight and length distribution for the studied age group. Researchers have highlighted the importance of studying EBF children to build appropriate reference growth curves [27].

Furthermore, although the infants evaluated in the present study came from a single geographical area, the sample size was predetermined according to the proposed goal, which increased the possibility of identifying differences between the comparison curves. However, this study was not intended to be a population study or to be representative of the Brazilian population. Thus, the extrapolation of its results to other populations should be conducted with caution [28].

The results of the present study were similar or superior to the reference curves [3, 8], which did not require EBF throughout the first six months of life as inclusion criteria. Additionally, our study’s findings are similar to those of other researchers who studied EBF children in this age group [23, 24, 29].

## Conclusion

Exclusive breastfeeding in the first six months of life provides adequate physical growth and results in weight and height gains that are similar to or greater than those depicted in the NCHS, 1997 and WHO, 2006 curves.

The superior weight means in the fifth and sixth months of life among the children in our study suggest that the growth curves of EBF children during the second quarter of life were greater than those of children who received other forms of feeding.

Finally, the official recommendations of WHO and the American Academy of Pediatrics were reinforced by our findings, as they recommend exclusive breastfeeding during the first six months of life as the ideal feeding strategy to ensure the healthy physical growth of infants in this age group [6, 30].
